# A locking compression plate as an external fixator for treating infected nonunion of the humeral diaphysis

**DOI:** 10.1186/s12893-016-0167-9

**Published:** 2016-08-05

**Authors:** Cong Xiao, Fan Tang, Yong Zhou, Wenli Zhang, Yi Luo, Hong Duan, Chongqi Tu

**Affiliations:** Department of Orthopedics, West China Hospital, No.37 Guoxue Xiang, Chengdu, Sichuan 610041 Peoples’ Republic of China

**Keywords:** Nonunion, Humeral diaphysis, Infection, External fixator, Plate

## Abstract

**Background:**

Infected nonunion of the humeral diaphysis is a challenging problem for orthopedic surgeons. This study aimed to evaluate the outcome of using a locking compression plate (LCP) as a definitive external fixator in the management of infected nonunion of the humeral diaphysis after failure of internal fixation.

**Methods:**

We retrospectively reviewed a series of seven patients with infected nonunion of the humeral diaphysis treated with an LCP as an external fixator between June 2010 and August 2014. There were five males and two females, with an average age of 40.9 years. Six out of seven patients had been definitively diagnosed with infection due to known bacteria by germiculture. The clinical and radiographic outcomes were retrospectively evaluated.

**Results:**

All patients were followed-up for a mean period of 26.3 months (range 12–48 months). All fractures obtained complete bone union, and the average time to bone union was 7.9 months (range 3.5–15 months). All infections were eventually resolved without any recurrence of deep infection. Pin tract infection was only seen in one case. Only one patient had transient radial nerve palsy after surgery for traction. The average shortening length of the affected upper limb was 3 cm (range 2–4 cm) compared with the contralateral limb. At the last follow-up, the average Disabilities of the Arm, Shoulder and Hand score of the involved limbs was 3.2 (range 0–13.4). All patients obtained excellent or good functional results, and returned to their original work.

**Conclusions:**

The novel use of an LCP as a definitive external fixator was an effective method for treating infected nonunion of the humeral diaphysis. However, a large-scale prospective clinical study is still needed to verify these findings.

## Background

Nonunion following operative treatment of humeral diaphyseal fracture occurs in approximately 2.5–13 % of cases [1]. However, infected nonunion of the humeral diaphysis following failed internal fixation is rare, and poses a challenging problem for orthopedic surgeons; this condition can cause problems including multiple sinuses, osteomyelitis, bone and soft tissue loss, osteopenia, adjacent joint stiffness, complex deformities, and multidrug-resistant polybacterial infection [2]. Traditional modalities for treating infected nonunion involve thorough debridement, implantation of antibiotic-containing cement chains/rod, and bone grafting or vascularized bone flap transferal, as well as the application of external fixator devices [1, 3, 4]. External fixation, especially the Ilizarov technique, is often used as a temporary or definitive adjunct for restoring bony stability to help eradicate the infection, and has been proven effective in the treatment of nonunion of the humeral diaphysis [5–7]. However, traditional external fixations are often bulky, uncomfortable, and inconvenient for the patient, typically leading to problems with sleeping and clothing, and causing impediment during daily activities.

A locking compression plate (LCP) has recently been introduced as an alternative external fixator, and has manifested satisfactory outcomes when used in open/closed tibial fracture and infected nonunion of the tibia or clavicle, overcoming the shortcomings of traditional external fixators [8–18]. This LCP technique has proved to be versatile, low profile, and well tolerated by patients, and has been encouraged as a useful adjunct in the treatment of complex reconstructive cases [9, 15]. However, to our knowledge, there has been no such research regarding use of an LCP as external fixation for infected nonunion of the humerus. The present study aimed to evaluate the outcome of using an LCP as an external fixator for treating infected nonunion of the humeral diaphysis after failure of internal fixation.

## Methods

### Patients

Between June 2010 and August 2014, a total of seven patients with infected nonunion of the humeral diaphysis were treated at our department using an LCP as an external fixation. Cases without infection and those with infected nonunion of proximal or distal humeral fracture were excluded from this study. The average age of the patients at presentation was 40.9 years (range 26–61 years). Five patients were male and two were female. Of these seven cases, six were closed humeral diaphyseal fractures, and one was an open fracture (Gustilo grade II). The AO/OTA classification of these patients is listed in Table 1. One case was accompanied by radial nerve injury. The initial operations of all patients (open reduction and internal fixation) were carried out in their respective local hospitals. The anterolateral approach was applied in six out of seven patients; the posterior approach was used in the case accompanied by radial nerve injury, which was repaired by end-to-end anastomosis. Erythema, swelling, purulent discharge of the affected upper limb, and implant failure were observed in all patients before revision surgery. Six out of seven patients presented with definite infection caused by known bacteria as diagnosed by germiculture. The mean time from fracture to revision surgery was 7 months (range 3–15 months). The patient data are described in Table 1.

**Table 1:** Patient demographics

Case	Age(y)/Gender	Open/Closed frcatue	AO/OTA classification	Gustilo grade	Initial treatment	Initial approach	Radial nerve injury	Time from fracture to revision surgery (months)	Type of organism
1	39/M	closed	12-A2	─	ORIF	anterolateral	no	7.2	Staphylococcus aureus
2	40/F	closed	12-A3	─	ORIF	posterior	yes	3	Enterobacter cloacae
3	37/M	closed	12-B2	─	ORIF	anterolateral	no	4.5	Staphylococcus aureus
4	37/M	closed	12-B2	─	ORIF	anterolateral	no	6	No growth
5	26/F	open	12-B1	II	Debridement and ORIF	anterolateral	no	6	MRSA
6	61/M	closed	12-B3	─	ORIF	anterolateral	no	15	Pseudomonas aeruginosa
7	46/M	closed	12-C1	─	ORIF	anterolateral	no	7.5	Staphylococcus aureus

*M* Man, *F* female, *ORIF* Open reduction and internal fixation, *MRSA* Methicillin-resistant Staphylococcus aureus

All patients were retrospectively evaluated clinically and radiographically. The functional results were evaluated according to the Disabilities of the Arm, Shoulder and Hand (DASH) scoring system [19]. Complications evaluated included nonunion, deep infection, pin tract infection, implant failure, and limb shortening. Radiographs were obtained at regular intervals: at the time of admission, immediately postoperatively, at postoperative 1, 3, 6, and 12 months, and at the final follow-up. Union was defined as the presence of a mature, bridging callus of three to four cortices seen on radiography, with the simultaneous absence of implant loosening or breakage and absence of pain during weight-bearing. Nonunion was defined as the absence of radiological signs of union 9 months postoperatively, without any tendency toward progressive union. Deep infection was defined according to clinical symptoms (such as erythema, swelling, and presence of purulent discharge) and laboratory examination results (such as total leukocyte count, C-reactive protein level, and erythrocyte sedimentation rate).

### Surgical technique

Under general or regional anesthesia, six of the seven patients were placed in the supine position with an arm table, and one patient was placed in the contralateral decubitus position because the posterior approach was used in the first surgery. The involved limb was prepared and draped in the usual standard sterile fashion, and the quondam incision was chosen (anterolateral in six cases, and posterior in one). All surgeries were performed by the same orthopedic surgeon (Chongqi Tu). The radial nerve was first explored and marked with a catheter. Care was taken not to damage the radial nerve during the entire procedure. In cases with an LCP still in situ, the LCP was removed. A thorough debridement was then performed, including resection of avascular bone, excision of sinus tracts and infected scarred soft tissue, collection of representative tissue culture samples, and opening of the medullary canal using a drill. Cortical bleeding was taken as an acceptable sign of vital tissue procurement [20]. After complete debridement, the nonunion site was reduced and aligned. Both fracture ends were pruned for better reduction and bony contact, regardless of the occurrence of bone defection. The fracture site was stabilized using temporarily placed Kirschner wires or 3.5 mm screws; otherwise, reduction was temporarily maintained by the assistant with two Kocher clamps.

**Fig. 1:**
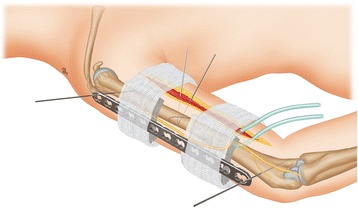
Schematic diagram of external plate placement

Once the fracture was acceptably reduced, an LCP was applied as an external fixator. A suitable LCP position was carefully considered before implanting screws. The LCP was placed over the anterolateral side in the six patients that were operated on via the anterolateral approach, whereas the LCP was placed over the posterior side in the patient in whom the posterior approach was used. To ensure that the plate was matched to the bone, we performed temporary positioning by drilling one 2.0 mm Kirschner wire into the cortex of the shaft at the most proximal hole of the LCP and another similarly at the most distal hole. A stack of evenly folded towels was then provisionally placed as a spacer under the plate, separating the plate from the skin (Fig. 1). To optimize mechanical stability, the LCP was placed as close to the bone as possible, while still allowing for potential swelling of the soft tissue. Because mechanical stability decreases as the distance between the plate and bone increases [16, 21], we chose relatively long plates with at least three 4.5 mm screw holes on both sides of the fracture site, except in cases where the salvaged LCP was reused. Generally, for the patients with an LCP in situ, the removed LCP was soaked in a container with povidone iodine for more than 30 minutes and then reused as the external fixator, with new screws being applied. For those without an LCP in situ, a 4.5 mm LCP (Weigao Orthopaedic Device Co., Ltd., Shandong, China) was applied as the external fixator. The first screw was implanted in the most distal hole, ensuring bicortical fixation was achieved; extreme care was taken when implanting the distal screws. We operated carefully under direct vision to avoid damaging the radial nerve. The second screw was then implanted in the most proximal hole. When alignment was deemed satisfactory fluoroscopically, the remaining screws were implanted in the usual fashion. The position and orientation of screws were reassessed under fluoroscopy.

### Postoperative management

Screw tracts were sterilized with iodophor or 75 % alcohol three times per day. Intravenous antibiotics were administered for 3–4 weeks according to antimicrobial susceptibility testing for tissue specimens or pus harvested intraoperatively (vancomycin in one case; cefoperazone sodium and tazobactam sodium in six cases); no bacterial growth was detected in one case. Third-generation cephalosporin oral medication was then administered for 4–6 weeks. The patients were encouraged to perform initial pendulum and elbow flexion-extension exercises from postoperative day 2. Functional use of the limb for light tasks was allowed when patients could tolerate it, usually from 6 weeks postoperatively.

## Results

**Fig. 2:**
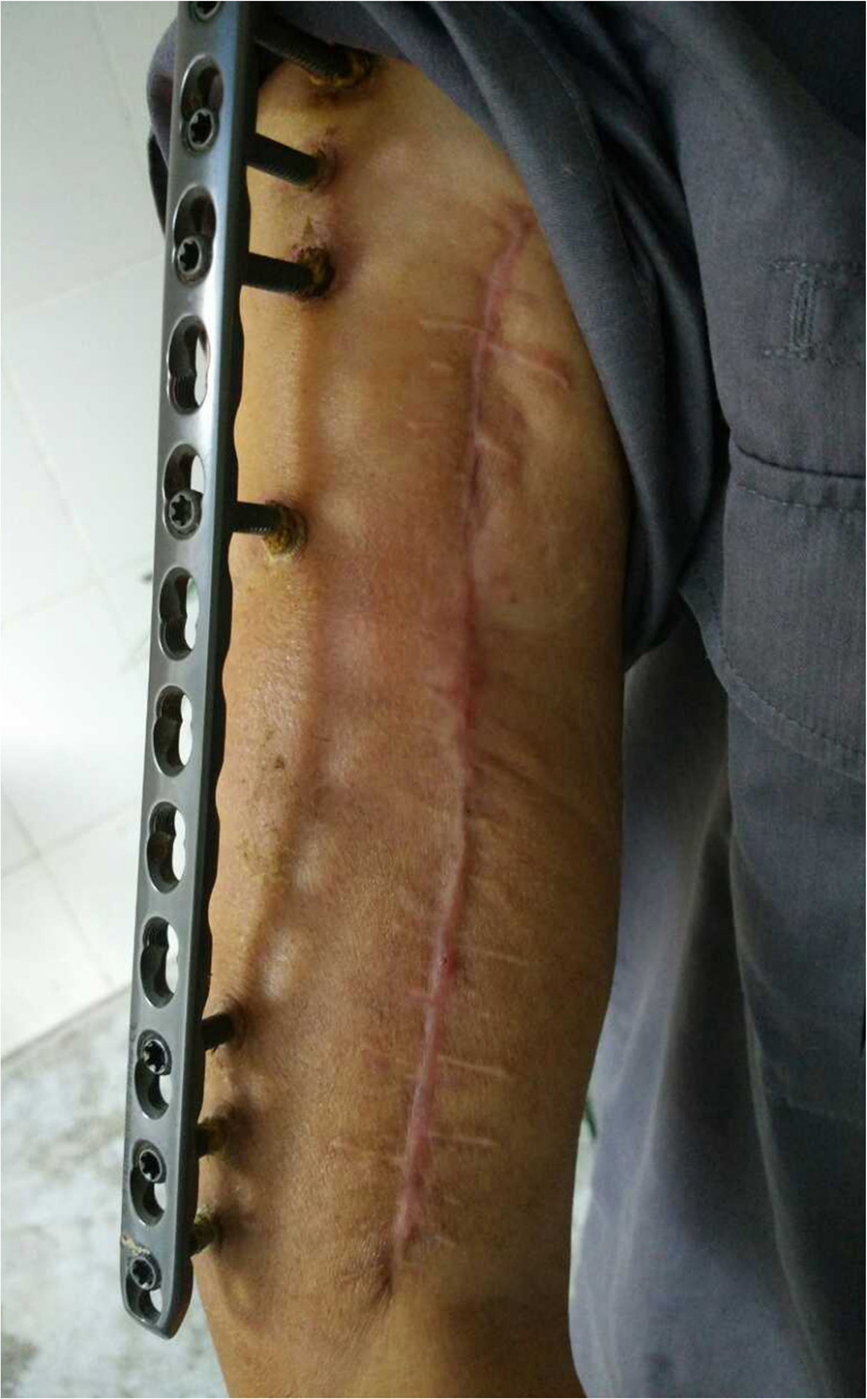
Appearance of external fixation of LCP showing good skin adherence to the fully threaded titanium screws

**Fig. 3:**
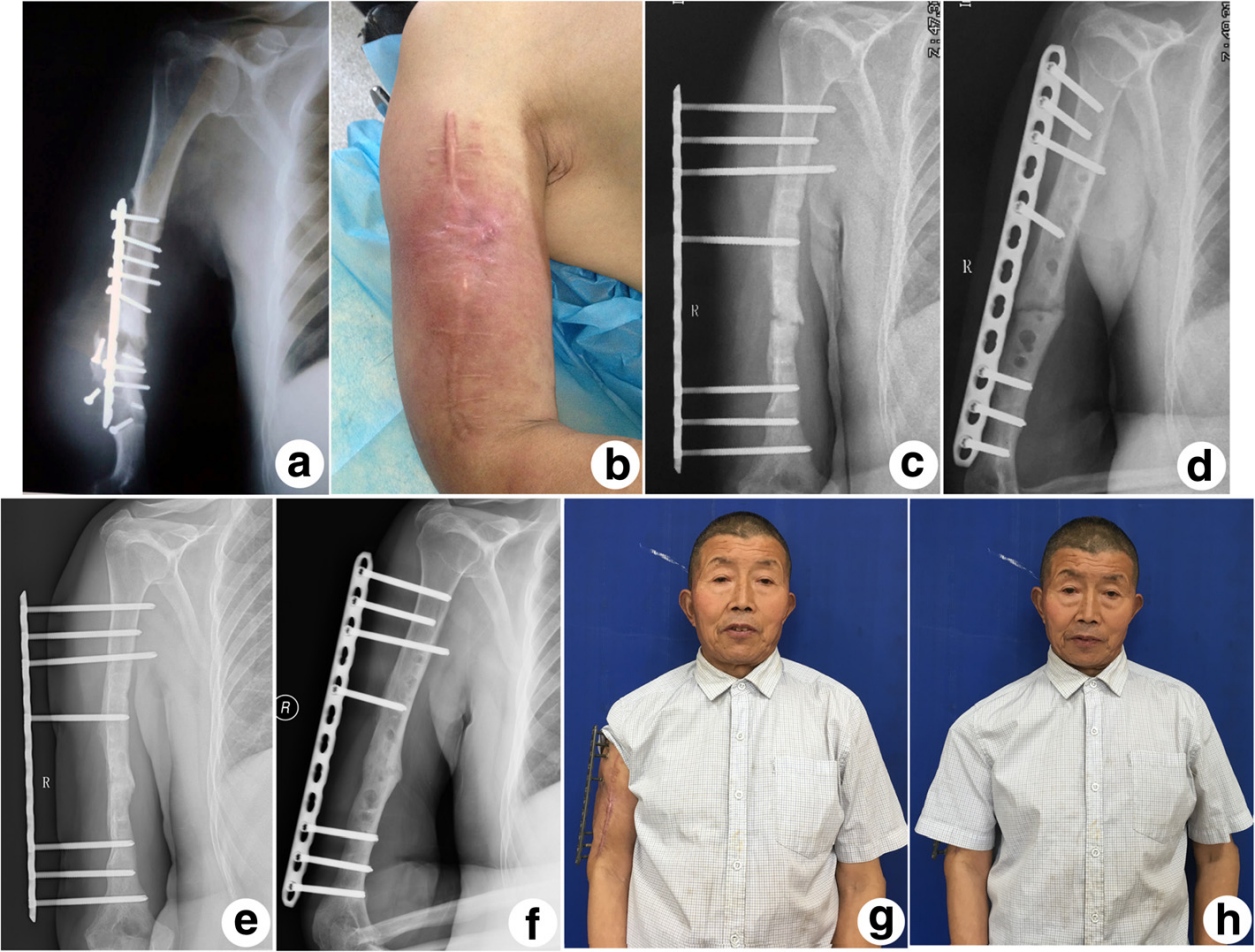
(**a**) X-ray of A 61-year-old male showing implant failure, nonunion and soft tissue swelling at the 15-month after the first surgery. (**b**) Extensive soft tissue swelling and erythema occurred in the affected upper limb. (**c**) Anteroposterior and (**d**) lateral X-ray at one month after revision surgery. (**e**) Anteroposterior and (**f**) lateral X-ray at the 6-month follow-up showing bony healing without any implant failure. (**g**) and (**h**) In situ plate showing low profile and well concealed under clothing

**Fig. 4:**
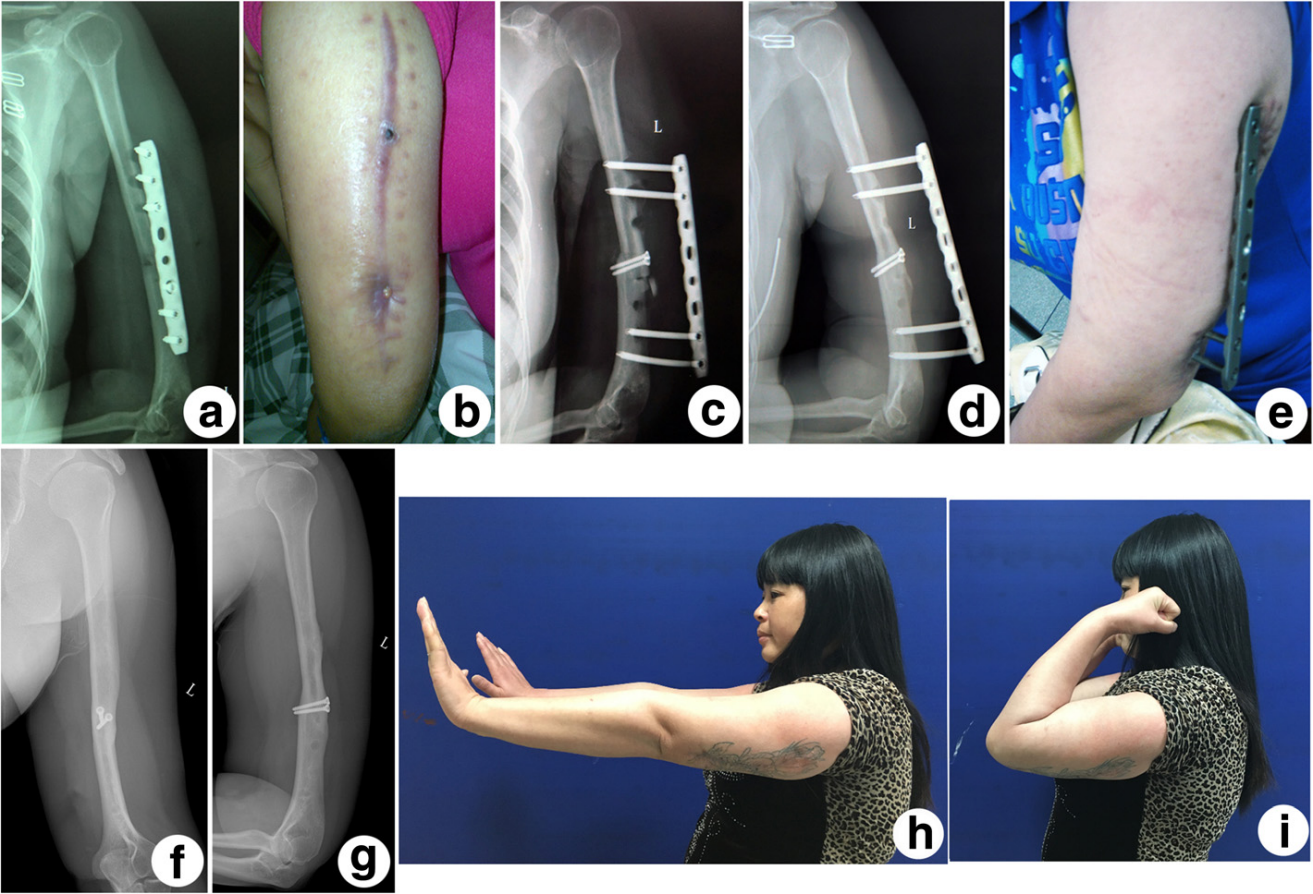
(**a**) X-ray of A 40-year-old female showing implant failure, nonunion at the 3-month after the first surgery. (**b**) Erythema, sinus and purulent discharge were seen in the affected upper limb. (**c**) Immediately postoperative X-ray showed the quondam LCP was applied as external fixator. (**d**) X-ray showing complete bone union at the 4-month follow-up. (**e**) Photograph showing the low profile plate. (**f**) Anteroposterior and (**g**) lateral X-ray at the last follow-up showing a full bony healing with an acceptable alignment. (**h**) and (**i**) Photographs at the last follow-up showing excellent function of the elbow, recovery of radial nerve function and absence of erythema, sinus or purulent discharge of the affected upper limb

All patients were followed-up for a mean of 26.3 months (range 12–48 months). All fractures obtained complete bone union in an acceptable position. The plate was removed in the outpatient clinic when full bony healing was observed. The average bone union time after revision surgery was 7.9 months (range 3.5–15 months). All infections were eventually resolved without any recurrence of deep infection. No loosening or failure of the implant was observed. The skin appeared to grow onto the fully-threaded titanium screws in all cases (Fig. 2). The symptoms of radial nerve injury in case No. 2 had resolved by 6 months after the initial trauma. Pin tract infection was seen in only one case at postoperative week 4, which resolved with intensive care of the screw site. One patient without preoperative radial nerve injury presented with transient radial nerve palsy after surgery for traction; this spontaneously resolved within 2 months with conservative treatment. The average shortening of the affected upper limb compared with the contralateral limb was 3 cm (range 2–4 cm). At the last follow-up, the mean range of motion (ROM) of the elbow was 1.4° extension and 131.4° flexion. The average DASH score of the involved limbs was 3.2 (range 0–13.4). All patients obtained excellent or good functional results and returned to their original work. The results of the study are summarized in Table 2. Typical cases (case 2 and 6) are shown in Figs. 3 and 4.

**Table 2:** Patient results

Case	Follow-up time(months)	Union time after revision surgery(months)	DASH scores at the last follow-up	Complications	Limb shortening(cm)	ROM of elbow(degree)	
						Flexion	Extension
1	28	8.5	1.7	none	3.5	130	0
2	48	4	0	none	4	140	0
3	32	10	0	none	3.5	135	0
4	20	3.5	2.5	transient radial nerve palsy	2	125	0
5	30	15	3.3	Pin tract infection	2.5	135	0
6	14	6	13.4	none	2.5	125	10
7	12	8	1.7	none	3	130	0

## Discussion

In this study, we reported seven cases of infected nonunion of the humeral diaphysis successfully treated in a one-stage procedure using an LCP as an external fixator.

Infected nonunion of the humerus is rare, and these cases are challenging to treat [1, 3, 4]. Treatment generally consists of a two-stage procedure. The first procedure involves removal of the previous implant, thorough debridement with collection of deep tissue for culture and exposure of fresh bleeding bone ends with sequestrectomy of the nonunion site, and application of an external fixator. Definitive internal fixation is performed in the second stage after eradication of infection.

LCPs have recently been used as a substitute for traditional external fixators, and have proved to be a highly popular alternative in the management of open fracture [12–16], infected nonunion [8–10, 17, 22], and even closed fracture of the tibia [11, 23, 24]. Locking screws can lock directly into the plate to obtain a stable connection instead of relying on friction between the plate and the bone, which is similar to the principle of external fixation. The LCP as an external fixator was first advocated by Kloen [9], who called this technique “supercutaneous plating”. To our knowledge, no other reports have described the technique of using an LCP as an external fixator for the treatment of infected nonunion of the humeral diaphysis.

The main advantage of external application of an LCP is the ability to construct a low profile frame. We believe that this makes it more suitable for management of infected nonunion of upper limb bones such as the humerus. Upper limbs have a more nimble motion than lower limbs; hence, when a standard external fixator is applied, the bulkiness and sharp edges of the device cause inconvenience during daily activities. In contrast, external application of an LCP in the humerus can allow more comfortable early functional exercise because of its low profile frame achieved by contouring the plate close to the skin; it can also be well concealed under clothing, making it more acceptable to patients.

External fixation of an LCP also results in less pin site problems. Bassiony et al. [1] reported that pin tract infections were seen in four of eight patients (50 %) who underwent traditional external fixation for humeral fracture. In the present study, pin tract infection was only seen in one screw of one patient. We attribute this extraordinarily low rate of pin tract infection to fully-threaded titanium screws that had better biological compatibility and adhered more easily to the skin compared with the partially-threaded stainless steel Schanz screws used in traditional external fixation [9, 11].

The use of an LCP as a definitive external fixator did not seem to adversely affect bone healing. We note that an LCP is usually only applied as a temporary external fixation [9, 15]; after resolution of the infection or healing of the wound, definitive internal fixation is generally performed, probably due to concerns regarding the potentially insufficient strength of an external locking plate. Kanchanomai et al. [21] designed a biomechanical test of tibial fracture externally fixed with an LCP, and reported that an increased distance between the bone and the implant significantly decreased the construct stability; however, all models were cyclically loaded beyond 500,000 cycles without any failure of the LCP [21], and so failure of the LCP is unlikely to be a critical issue in clinical cases. This is supported by previous research; one study reported that eight open tibial fractures healed after only first-stage treatment due to patients’ refusal of second-stage treatment [16], a series of 12 tibial injuries treated using an LCP as a definitive fixator resulted in union with no loosening or failure of implant in all cases [12], and 31 patients with infected nonunion or open fracture mainly of the upper extremity treated using an AO-plate as an definitive external fixator (via the same principle as an LCP) resulted in healing of both the infection and the nonunion [22]. Similarly, in the present study, the outcome was satisfactory in all seven cases of infected nonunion of the humeral diaphysis treated with an LCP applied as definitive external fixation.

Using an LCP as definitive external fixation may be cheaper than traditional treatment. When traditional monoaxial external fixators are used, the pin can easily loosen several months after surgery, prompting surgeons to apply it only temporarily in the first stage, and perform definitive internal fixation in the second stage. In contrast, failure of an LCP is unlikely to be a critical issue for clinical cases [21]. External application of an LCP may afford enough stabilization until fracture union is observed; however, if nonunion of the fracture occurred, then internal fixation and bone grafting would be needed. We believe that performing extensive debridement and adequate pruning of the fracture ends can improve the fracture healing rate. In our study, we elected not to attempt preservation of the length of the humeral shaft, as a loss of less than 3–4 cm in the upper extremity is generally well tolerated by patients [25]. Therefore, extensive debridement was conducted and oblique or Z-shaped contact surfaces were pruned on both sides of the fracture for better reduction and bony contact, giving an average shortening length of the affected upper limb of 3 cm. As a result, bone union was seen in all patients after a one-stage procedure. Hence, the second operation for exchanging definitive internal fixation was avoided and the total costs were dramatically decreased compared with traditional two-stage therapy for infected nonunion. The cost was further decreased in three of the cases in this series, as the old plates were salvaged, sterilized with povidone iodine, and then externally reused.

Surgery involving LCP fixation is technically more difficult than traditional external fixation. First, unlike traditional fixation in which half-pins are implanted prior to cross-bar connection, acceptable reduction of the fracture must be achieved before application of the plate; the plate is only able to move in one plane once one screw is placed. Second, accurate screw placement remains relatively difficult due to subtle shifts of the plate, leading to great deviations at the level of bone. Therefore, to achieve as much bicortical fixation as possible, the two Kirschner wires were temporarily placed over the most proximal and distal holes of the plate to penetrate the bicortex of the bone, so that the plate was matched to the bone. When lateral placement of the plate is applied, implanting of the distal screws is relatively difficult due to the special geometry of the distal humeral shaft and its position adjacent to the radial nerve. Operation under direct vision is essential, and the first screw should be placed over the most distal hole of the plate to achieve bicortical fixation, as bicortical fixation is relatively easy to obtain at the proximal fragment. Third, alignment of the bone should be reassessed after placement of the first screw, as there could potentially be displacement of the fracture caused by loosening of the Kirschner wires or fatigue of the assistant. As long as two screws are implanted, the plate position does not alter. Adjustment of the plate position may sacrifice the drilled bone holes, leading to increased difficulty of bicortical engagement; cases with only unicortical purchase have 50 % less rigidity than bicortical configurations [26].

There were several limitations of our study. First, the number of cases was relatively small and there was no control group. The small sample size may have led to deviation over the results of bone healing in all cases; it still remains controversial whether an LCP, originally designed for internal fixation, can be applied as an external fixator. Furthermore, reusing the old plate seems unacceptable; however, the poor economic situation of some patients forced us to choose this method, and this proved to be a feasible strategy in our study.

## Conclusions

The low-profile LCPs used as external fixators for infected nonunion of the humeral diaphysis gave patients a comfortable clinical experience, provided adequate stability until bone union, and could be removed without difficulty in the outpatient clinic. There were relatively low overall costs because there was no need for a second operation to remove the plate, and the old plates were reused in some cases. A large-scale prospective clinical study is warranted to verify our results. Nevertheless, the present study describes a useful alternative for treatment of this challenging condition.

## Abbreviations

DASH, disabilities of the arm, shoulder and hand; LCP, locking compression plate; ROM, range of motion
